# Comparison of Three Commercial ELISA Kits for Detection of Antibodies Against SARS-CoV-2 in Serum Samples from Different Animal Species

**DOI:** 10.3390/v17050716

**Published:** 2025-05-16

**Authors:** Leira Fernández-Bastit, Sílvia Marfil, Edwards Pradenas, Julià Blanco, Júlia Vergara-Alert, Joaquim Segalés

**Affiliations:** 1 Unitat Mixta d’Investigació IRTA-UAB en Sanitat Animal, Centre de Recerca en Sanitat Animal (CReSA), Campus de la Universitat Autònoma de Barcelona (UAB), 08193 Bellaterra, Spain; leirafernandezbastit@gmail.com; 2 IRTA, Animal Health, Centre de Recerca en Sanitat Animal (CReSA), Campus de la Universitat Autònoma de Barcelona (UAB), 08193 Bellaterra, Spain; 3 IrsiCaixa, 08916 Badalona, Spain; smarfil@irsicaixa.es (S.M.); epradenas@irsicaixa.es (E.P.); jblanco@irsicaixa.es (J.B.); 4 University of Vic-Central University of Catalonia (UVic-UCC), Vic, 08500 Barcelona, Spain; 5 Infectious Disease Networking Biomedical Research Center (CIBERINFEC), Carlos III Health Institute, 28029 Madrid, Spain; 6 Germans Trias i Pujol Research Institute (IGTP), Can Ruti Campus, 08916 Badalona, Spain; 7 Departament de Sanitat i Anatomia Animals, Facultat de Veterinària, Campus de la Universitat Autònoma de Barcelona (UAB), 08193 Bellaterra, Spain

**Keywords:** severe acute respiratory syndrome coronavirus 2 (SARS-CoV-2), humoral immune response, animal serology, ELISA, pseudovirus neutralization test (pVNT), one health, zoonosis

## Abstract

The severe acute respiratory syndrome coronavirus 2 (SARS-CoV-2) caused the coronavirus disease 19 (COVID-19) pandemic, significantly impacting global health, economies, and social stability. In February 2020, the first cases of SARS-CoV-2 infections in animals were documented, highlighting the potential risks posed by regular human–animal interactions in facilitating viral transmission. In consequence, it is essential to validate surveillance methods for SARS-CoV-2 in animals. In the present study, 101 sera from different animal species (36 cats, 41 dogs, 4 ferrets, 10 wild boar, 6 domestic goats, and 4 lions) were tested using three different ELISA kits to evaluate humoral responses against SARS-CoV-2. ELISA results were compared and correlated with a pseudovirus neutralization test (pVNT), considered as the reference assay. ELISA-1, targeting the receptor binding domain (RBD) neutralizing antibodies (nAbs) of SARS-CoV-2, exhibited the highest diagnostic performance, and proved to be a reliable tool for initial screenings in high-throughput animal studies. In contrast, ELISA-2 (also targeting RBD nAbs) and ELISA-3 (targeting nucleoprotein antibodies) demonstrated lower sensitivity for detecting seropositive animals.

## 1. Introduction

The most probable zoonotic origin of the severe acute respiratory syndrome coronavirus 2 (SARS-CoV-2), together with ongoing reports of infections in various animal species, underscore the critical need for sustained surveillance studies in animal populations [1]. Regarding emerging zoonotic diseases, including coronavirus disease 2019 (COVID-19), serological tests are invaluable tools for detecting exposure to the infectious agent, identifying susceptible species, and recognizing potential animal reservoirs [2].

ELISA is a widely used serological assay that offers a cost-effective, time-efficient means for high-throughput analysis [3,4]. More complex but also more specific and sensitive techniques, including the pseudovirus neutralization test (pVNT), virus neutralization test (VNT), and plaque reduction neutralization test (PRNT), are essential to confirm initial ELISA screening results [4]. pVNT uses pseudoviruses, which are viral particles coated with a heterologous envelope (E) protein responsible for entry to cells [5]. This assay allows for assessing the functional capability of antibodies to neutralize the virus by blocking its entry into cells and preventing further infection. pVNT relies on infectious pseudoviruses and live cells, which better simulates live virus entry and infection compared to ELISA [4,5]. The latter does not evaluate the functional capacity of antibodies but the capacity of them to bind purified recombinant proteins of the virus. Accordingly, pVNT highly correlates with VNT and PRNT, both of which measure neutralizing antibody (nAb) titers using infectious viruses [5,6]. Unlike VNT and PRNT, which require biosafety level 3 (BSL-3) facilities when studying highly pathogenic viruses, pVNT can be performed in BSL-2 laboratories, as pseudoviruses lack autonomous replication and can infect cells only in a single cycle [4,5].

In COVID-19 patients and SARS-CoV-2-infected animals, most nAbs target the receptor binding domain (RBD) of the spike (S) glycoprotein (the sole exposed viral protein on the virion), while antibodies against the inner nucleocapsid (N) protein antibodies are primarily non-neutralizing binding antibodies [7]. RBD-targeting nAbs are associated with protective immunity in humans and some animal species by blocking viral entry and subsequent infection [8,9,10,11,12]. Antibodies against N protein, on the other hand, have been recognized to mitigate severe COVID-19 in human patients and promote the resolution of infection by enhancing immune responses that facilitate the clearance of viral particles, toxins, and infected cells [13,14,15]. Importantly, the N encoding gene is highly conserved among coronaviruses (CoVs), leading to potential cross-reactivity in serological assays [13,16]. Meanwhile, the RBD genomic sequence is highly variable among CoVs, providing greater specificity for SARS-CoV-2 detection [16,17,18]. This is of particular importance to consider when developing serological assays for animal samples, given that numerous other CoVs have the capability to infect a wide range of animal species [19]. Moreover, most commercial ELISA kits used for analyzing animal samples were developed and validated exclusively using human samples, not animal ones [18,20]. Hence, it is noteworthy that the interpretation of results may vary between humans and animal samples, and even among samples from different animal species. So far, various commercial and in-house serological assays have been employed worldwide to detect SARS-CoV-2 antibodies in animals [18,20,21,22].

The main objective of this study was to evaluate and compare the performance of three commercial ELISA kits for the detection of SARS-CoV-2 antibodies in serum samples from multiple animal species. By evaluating the diagnostic performance of these three different ELISA kits against pVNT as reference method, the most optimal ELISA assay for high-throughput screening in epidemiological studies involving large numbers of animal samples was proposed.

## 2. Materials and Methods

### 2.1. Study Design

A total of 101 serum samples were included in the present comparison study. Samples corresponded to different animal species: 36 domestic cats (*Felis catus*), 41 dogs (*Canis familiaris*), 4 ferrets (*Mustela putorius furo*), 10 wild boar (*Sus scrofa*), 6 domestic goats (*Capra aegagrus hircus*), and 4 lions (*Panthera leo*) (Table S1).

SARS-CoV-2 acute infection was previously assessed by RT-qPCR in all animals [23,24,25,26], except for wild boar, since Suidae are not considered susceptible to this virus [27]. One dog (Dog 36 in Table S1), all experimentally infected goats, and all lions (Table S1) tested positive [23,24,26]. Additional information, including animal species, date of blood sampling, predominant SARS-CoV-2 variant at the sampling period, and the results of the RT-qPCR, is summarized within the Table. From the experimental study in domestic goats [26], three animals sampled at 2 days post-inoculation (dpi) and three animals sampled at 18 dpi were included in the present study.

Herein, serum samples were analyzed by ELISA-1, ELISA-2, ELISA-3, and pVNT (used as a reference) to assess the sensitivity and specificity of each ELISA kit. The diagnostic performance of each assay was also evaluated according to the cut-off established for the pVNT. Due to limited volumes, not all the samples were tested by each serological test (Table S1).

### 2.2. Antibody Detection Tests

Three different commercial ELISAs kits were used to detect antibodies against the most immunogenic antigens of SARS-CoV-2: the RBD and the N proteins [13,28]. Both, the cPass SARS-CoV-2 Neutralization Antibody detection kit (Genscript, The Netherlands; ELISA-1) and the SARS-CoV-2 NeutraLISA kit (EUROIMMUNE, Germany, ELISA-2) are competitive ELISA-based assays that detect nAbs against the RBD. Both kits use purified recombinant RBD and human angiotensin converting-enzyme 2 (hACE2) proteins. The ID Screen^®^ SARS-CoV-2 Double Antigen Multi-species assay (IDVET, France; ELISA-3) detects total antibodies against the N protein of SARS-CoV-2. In addition, a pVNT based on pseudoviruses derived from the modified HIV, engineered to express the S glycoprotein of SARS-CoV-2 along with a luciferase reporter gene, was used as previously described [6].

#### 2.2.1. ELISA-1: cPass SARS-CoV-2 Neutralization Antibody Detection Kit

All samples (n = 101) were evaluated by ELISA-1. The protocol was performed following the manufacturer’s instructions, as previously described [25]. Results were expressed by the formula provided by the manufacturer’s protocol: % Signal Inhibition (%IH) = (1 − (OD value of sample/OD value of negative control)) × 100%. Samples with %IH ≥ 30% were considered positive for RBD nAbs.

This test is based on the RBD of the ancestral (B.1) SARS-CoV-2 first detected in Wuhan (China). ELISA-1 was validated using human samples from the United States from early stages of the pandemic (from March 2020 to November 2020), when other circulating variants had still not emerged [29].

#### 2.2.2. ELISA-2: SARS-CoV-2 NeutraLISA Kit

A total of 87 out of 101 samples were tested by ELISA-2 (Table S1) following the manufacturer’s instructions and as previously described [30]. Results were expressed as an inhibition percentage (%IH) according to the formula provided by the manufacturer’s instructions: %IH = 100% − (sample OD × 100%/mean OD of black controls). An %IH ≥ 35% was considered positive neutralization, while %IH ≥ 20 to <35 was considered doubtful, and %IH < 20 was considered as a negative neutralization.

This test provides S1/RBD from the B.1 ancestral SARS-CoV-2. It was validated using human samples (manufacturer’s data); therefore, it is recommended for serum and plasma samples from humans.

#### 2.2.3. ELISA-3: ID Screen^®^ SARS-CoV-2 Double Antigen Multi-Species Assay

A total of 99 out of 101 samples were tested by the ELISA-3 (Table S1) following the manufacturer’s protocol and as previously described [30]. Results were analyzed by the following formula provided by the manufacturer’s protocol: sample/positive control (S/P)% = [(OD sample − OD negative control)/(OD positive control − OD negative control)] × 100. Samples with S/P% ≥ 60 were considered positive for N protein antibodies, S/P% from 50 to 60 were considered doubtful, and S/P% ≤ 50 were considered negative.

The use of this test is recommended for serum or plasma samples from cats, dogs, bovines, sheep, goats, horses, and any other susceptible animal species (manufacturer’s data).

### 2.3. Pseudovirus Neutralization Assay (pVNT)

All samples (n = 101) were tested by pVNT, following an already described protocol [25], with some modifications. Briefly, the HIV reporter pseudoviruses expressing the SARS-CoV-2 S protein from the ancestral virus (B.1 lineage) and luciferase were generated. Pseudoviruses expressing a VSV-G protein instead of the S protein were generated and used as a control of specificity, as previously described [6].

For the neutralization assay, 200 TCID50 (50% tissue culture infectious dose) of pseudovirus were pre-incubated with three-fold serial dilutions (from 1/60 to 1/14,580), of heat-inactivated sera samples, for 30 min at 37 °C. Next, human ACE2-overexpressing HEK293T cells were added onto mixed samples. After 48 h, cells were lysed with Britelite Plus Luciferase reagent (Perkin Elmer, Waltham, MA, USA) and luminescence was measured for 0.2 s with EnSight multimode plate reader (Perkin Elmer).

The neutralization capacity of the sera samples was calculated by comparing the experimental RLU from infected cells treated with each serum to the maximum RLUs (maximal infectivity calculated from infected untreated cells) and minimum RLUs (minimal infectivity calculated from uninfected cells) and expressed as percentage of neutralization: % Neutralization = (RLUmax − RLUexperimental)/(RLUmax − RLUmin) × 100. ID50 values were calculated by plotting and fitting neutralization values and the log of plasma dilution to a four-parameter equation in Prism 10.0.2 (GraphPad Software, San Diego, CA, USA). All ID50 (half-maximal inhibition dilution) values are reported as reciprocal dilution. Titers of nAbs equal or higher than 60 were considered positive, while those samples with lower titer (limit of detection: ID50 < 60) were considered negative.

### 2.4. Statistical Analyses

Sensitivity and specificity, and predictive positive and negative values (PPV and PNV, respectively) were calculated for each ELISA using pVNT as a reference (cut-off = ID50 60). The overall diagnostic performance of each test was determined using receiver operating curve (ROC) analysis and calculating the area under the curve (AUC).

A Spearman correlation analysis was conducted between each ELISA and pVNT, and among ELISAs detecting RBD nAbs (ELISA-1 and ELISA-2), and among ELISA-1 and ELISA-3, to study the correlation between the presence of RBD nAbs and N protein antibodies. Correlation analyses with *p*-values < 0.05 were considered significant.

All statistical analyses were performed using GraphPad Prism (version 10.0.2).

## 3. Results

### 3.1. Detection of SARS-CoV-2 Humoral Response by pVNT

All samples (n = 101) were evaluated by pVNT using a pseudovirus containing the S glycoprotein of the initial SARS-CoV-2 (B.1 lineage) identified in Wuhan. A total of 33 out of 101 (32.7%) samples tested positive, with ID50 values ranging from 92.7 to 9570, and the remaining 68 (67.3%) samples tested negative (ID50 < 60; Supplementary Materials)

### 3.2. Detection of SARS-CoV-2 Humoral Response by ELISA-1, ELISA-2, and ELISA-3

**Figure 1:**
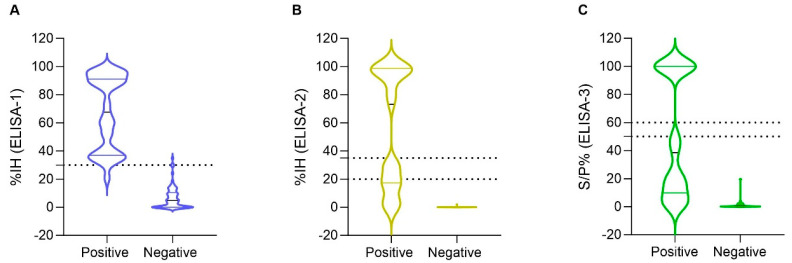
Distribution of inhibition (%IH) values of (**A**) ELISA-1 (n = 101), (**B**) ELISA-2 (n = 87) and sample/positive % (S/P%) values of (**C**) ELISA-3 (n = 99) among pVNT-positive and pVNT-negative results (X axes). Dashed lines indicate the cut-off for each serological assay. Negative values were represented as zero, and values exceeding 100 were represented as 100 for illustrative purposes. The values of pVNT-positive samples exhibited a wider distribution within ELISA-2 and ELISA-3, compared to ELISA-1. Two false-positive results were observed by ELISA-1, but none by ELISA-2 and ELISA-3.

All samples (n = 101) that were tested by pVNT were also evaluated by ELISA-1. From 33 pVNT-positive samples, 32 (96.9%) were positive (%IH ≥ 30) and 1 (3.0%) was negative by ELISA-1 (Figure 1A). However, from 68 pVNT-negative samples, 2 (2.9%) sera samples tested positive by ELISA-1. These samples corresponded to two wild boar that exhibited %IH values of 34.9% and 30.0%, respectively (Supplementary Materials).

In addition, a total of 87 samples out of 101 pVNT-evaluated samples were also tested by ELISA-2; non-tested sera were those with insufficient sera amount for testing. From 29 pVNT-positive samples, 15 (51.7%) samples were positive (%IH ≥ 35), 4 (13.8%) samples were considered doubtful (%IH ≥20 to <35), and 10 (34.5%) samples were considered negative (%IH < 20) by ELISA-2 (Figure 1B). This is represented by a wide distribution of the %IH values of positive pVNT samples within the ELISA-2 compared to ELISA-1 (Figure 1A,B). No false positive results were obtained by ELISA-2 (Figure 1B).

Finally, a total of 99 out of 101 samples were tested by the ELISA-3. Although false positive results were not observed, 19 (61.3%) samples tested negative (S/P% ≤ 50) from a total of 31 pVNT-positive samples (Figure 1C). This test has a similar distribution of the pVNT-positive samples as ELISA-2 (Figure 1B,C).

**Figure 2:**
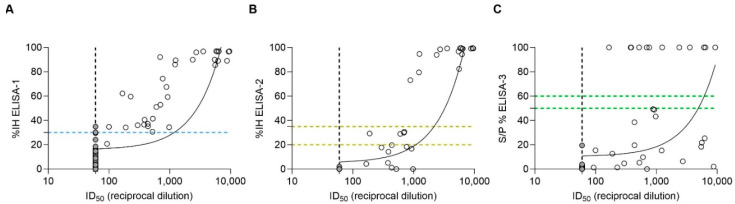
Spearman correlation analysis between pVNT and (**A**) ELISA-1 (r = 0.7985), (**B**) ELISA-2 (r = 0.7935), and (**C**) ELISA-3 (r = 0.6663). Dashed lines indicate the cut-off for each serological assay (blue color for ELISA-1; yellow color for ELISA-2; and green color for ELISA-3). Within ELISA assays, negative values were represented as zero, and values exceeding 100 were represented as 100 for illustrative purposes. ID50 (pVNT) is represented as the reciprocal dilution. Filled gray circles indicate samples out of the minimum limit of quantification of the pVNT.

In all ELISA tests, the higher the pVNT titer, the less likely occurrence of false negative results (Figure 2). Notably, pVNT-positive samples with titers <1000 were detected as doubtful or negative by ELISA-2. In parallel, all samples with pVNT titers ≥1000 were detected positive with IH% values ≥ 79% by ELISA-2 (Figure 2B). Meanwhile, ELISA-1 was able to detect positive samples at least from pVNT titers ≥ 60 (Figure 2A). In addition, all positive ELISA-3 samples that were also positive by pVNT gave values from S/P% > 100. In contrast, around two thirds of positive pVNT samples yielded negative results by ELISA-3 (Figure 2C).

A significant correlation (*p*-value < 0.05) was observed between each ELISA and pVNT, using Spearman correlation analysis. ELISA-1 showed a Spearman correlation coefficient (r) value of 0.7985 (%95 CI = [0.7118–0.8613]), ELISA-2 exhibited an r value of 0.7935 (%95 CI = [0.6966–0.8620]), and finally, ELISA-3 exhibited an r value of 0.6663 (%95 CI = [0.5357–0.77658]).

### 3.3. Qualitative Comparison of ELISAs with pVNT

To calculate sensitivity and specificity of each ELISA (ELISA-1, ELISA-2, and ELISA-3), the pVNT was used as the comparative reference technique with a cut-off = ID50 60. ELISA-1 exhibited the highest sensitivity (96.9%), followed by ELISA-2 (51.7%) and ELISA-3 (38.7%) (Table 1). When doubtful samples from ELISA-2 (n = 4) were considered positive, the sensitivity increased (65.5%), although this test still showed less sensitivity than ELISA-1, and higher sensitivity than ELISA-3. ELISA-1 showed the lowest specificity (97.1%), compared to ELISA-2 (100%) and ELISA-3 (100%). PPV and NPV are also represented in Table 1.

Serum samples from cats and dogs were also analyzed independently to assess the sensitivity and specificity of each ELISA for each species using pVNT as a reference (Table 2). Within cats, ELISA-1 exhibited the highest sensitivity (100.0%), followed by ELISA-2 (72.7%), and ELISA-3 (66.7%). When doubtful samples from ELISA-2 (n = 2) were considered positive, the sensitivity increased to 90.9%, although this test still showed less sensitivity than ELISA-1, and higher sensitivity than ELISA-3. Regarding dogs, ELISA-1 still exhibited the highest sensitivity (100.0%), followed by ELISA-3 (33.3%), and ELISA-2 (16.7%). The sensitivity of ELISA-2 increased to 33.3% when doubtful samples (n = 2) were considered positive. All tests showed a specificity of 100.0% for both cats and dogs. PPV and NPV are also represented in Table 2. All other species were not included in these analyses, since the number of samples per species was very low and/or samples were not analyzed with all the different assays.

**Figure 3:**
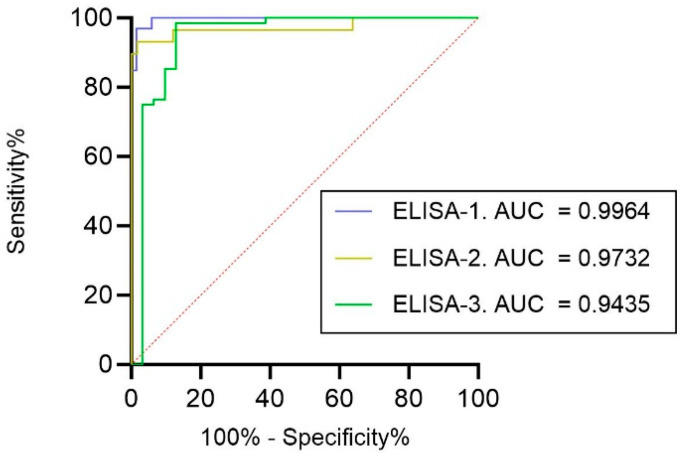
ROCs of all evaluated ELISAs using the pVNT as a reference (cut-off ID50 60). Associated AUC values are also represented.

For the overall diagnostic performance of ELISA-1, ELISA-2, and ELISA-3, ROC analyses were performed, and the AUC was calculated for each assay (Figure 3). Data were evaluated according to the cut-off established for the pVNT (ID50 60). The antibody assay with the highest AUC was ELISA-1 (AUC of 0.9964), followed by ELISA-2 (AUC of 0.9732) and ELISA-3, which exhibited the lowest AUC (0.9435).

### 3.4. Correlation of ELISAs Detecting RBD nAbs (ELISA-1 vs. ELISA-2)

**Figure 4:**
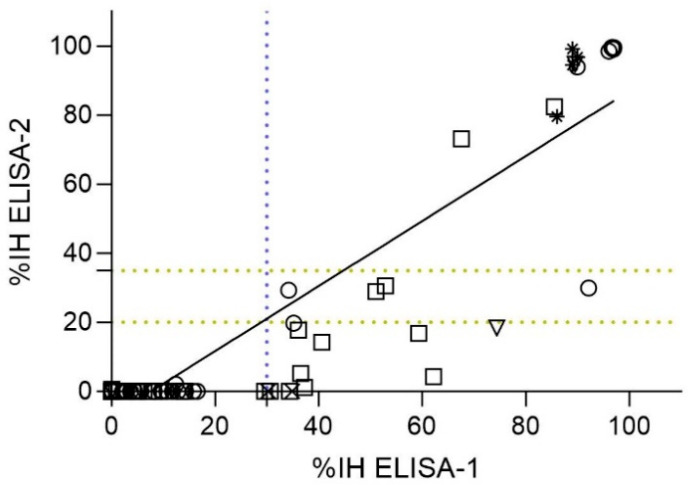
Spearman correlation analysis between ELISAs detecting nAbs against the RBD of SARS-CoV-2 (ELISA-1 and ELISA-2) (n = 87). Dashed lines indicate the cut-off for each serological assay (blue color for ELISA-1, and yellow color for ELISA-2). Negative values were represented as zero, and values exceeding 100 were represented as 100 for illustrative purposes. Species are indicated by different figures: circle (cats; n = 36), square (dogs; n = 41), downward triangle (ferret; n = 4), star (lions; n = 4), and cross (wild boar; n = 2). Spearman correlation coefficient value (r) = 0.6734 (95% CI = [0.5374–0.7767]).

A total of 87 out of 101 samples were analyzed by both ELISA-1 and ELISA-2, which similarly targeted RBD nAbs. A significant correlation (*p*-value < 0.05) between these assays (r = 0.6734; 95% CI = [0.5374–0.7767]) (Figure 4). Of the 56 samples that tested negative by ELISA-1, all tested negative by ELISA-2 (56/87; 64.37%). However, from 31 ELISA-1-positive samples (31/87; 35.63%), only 15 (15/31; 48.38%) samples tested positive (%IH ≥ 35%), 4 (4/31; 12.90%) samples tested doubtful (%IH ≥ 20 to <35), and 12 (12/31; 38.70%) samples tested negative (%IH < 20) by ELISA-2 (Figure 4). Generally, %IH values determined by ELISA-1 were slightly higher than those obtained by ELISA-2. Consequently, those %IH values close to the cut-off of ELISA-1 (%IH = 30%) were negative (%IH < 20) or doubtful (%IH ≥ 20 to <35) by ELISA-2 (Figure 4).

Samples from cats (n = 36) and dogs (n = 41) were also analyzed separately to investigate the correlation between ELISA-1 and ELISA-2 for each species. Significant correlation (*p*-value < 0.05) was observed for both species (r (Spearman) = 0.6625 [%95 CI = 0.4187–0.8173] for cats and r (Spearman) = 0.6143 [%95 CI = 0.3700–0.7791] for dogs). All samples from cats and dogs considered negative by ELISA-1 were also negative by ELISA-2 (25/36 cats [71.42%]; 29/41 dogs [51.22%]) (Figure 4). However, a total of 11 seropositive cats (11/36; 30.55%) were detected by ELISA-1, from which only 8 (8/11; 72.73%) cats tested positive, 2 (2/11; 18.18%) tested doubtful, and 1 (1/11; 9.09%) tested negative by ELISA-2. In addition, a total of 12 seropositive dogs (12/41; 29.26%) were detected by ELISA-1, from which only 2 (2/12; 16.66%) dogs tested positive, 2 (2/12; 16.66%) dog samples were doubtful, and 8 (8/12; 66.66%) dogs tested negative by ELISA-2 (Figure 4).

### 3.5. Qualitative and Quantitative Correlation of RBD nAbs (ELISA-1) and N Protein Antibodies (ELISA-3)

**Figure 5:**
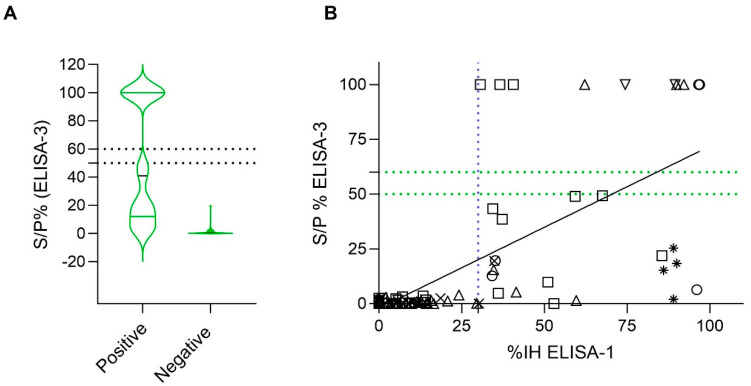
A qualitative and quantitative comparison analysis of ELISA-1 and ELISA-3 (N = 99). Negative values were represented as zero, and values exceeding 100 were represented as 100 for illustrative purposes. (**A**) Distribution of sample/positive % (S/P%) values of ELISA-3 within positive and negative ELISA-1 results (cut-off IH% ≥ 30). Discontinuous lines indicate the cut-off of ELISA-2. (**B**) Correlation analysis between ELISA-1 and ELISA-3. Dashed lines indicate the cut-off for each serological assay (blue color for ELISA-1, and green color for ELISA-3). Species are indicated by different figures: circle (cats; n = 34), square (dogs; n = 41), downward triangle (ferret; n = 4), upward triangle (goats; n = 6), star (lions; n = 4), and cross (wild boar; n = 10). Spearman correlation coefficient value (r) = 0.5999 (95% CI = [0.4518–0.7158]).

To determine the correlation between the presence of RBD nAbs and N protein antibodies, the correlation between ELISA-1, the most sensitive ELISA for detection of RBD nAbs, and ELISA-3 was evaluated (n = 99). All samples that were negative for the presence of RBD nAbs (67/99; 67.89%) were also negative for the presence of N protein antibodies (S/P% ≥ 60). However, from 32 (32/99; 33.3%) samples exhibiting RBD nAbs, only 12 (12/32; 37.5%) were positive for the presence of N protein antibodies (S/P% ≥ 60) (Figure 5A). The two ELISAs showed a significant (*p*-value < 0.05) correlation (r = 0.5999; 95% CI = [0.4518–0.7158]) (Figure 5B).

Samples from cats (n = 34) and dogs (n = 41) were analyzed separately to investigate the correlation between ELISA-1 and ELISA-3 for each species. Significant correlation (*p*-value < 0.05) was observed for both species (r (Spearman) = 0.5585 [%95 CI = 0.2620–0.7587] for cats and r (Spearman) = 0.5318 [%95 CI = 0.2593–0.7259] for dogs). All cats and dogs that were negative for nAbs RBD detection were also negative for N antibodies detection (25/34 cats [73.53%]; 29/41 dogs [51.22%]) (Figure 5B). However, from 9 (9/34; 26.47%) samples of cats exhibiting RBD nAbs (ELISA-1), 6 (6/9; 66.66%) were positive and 3 (3/9; 33.33%) were negative for N protein antibodies (ELISA-3). In addition, from 12 (12/41; 29.26%) positive dogs for RBD nAbs, only 4 (4/12; 33.33%) tested positive, and 8 (8/12; 66.66%) tested negative for N protein antibodies (ELISA-3) (Figure 5B).

### 3.6. Serological Assays Associated with RT-qPCR Detection in Animal Samples

For most animals included in this study, RT-qPCR was previously performed in each corresponding study, to assess whether they were suffering from a SARS-CoV-2 acute infection at the time of sampling. This includes all samples from pets (cats, dogs, and ferrets), although only one dog tested positive by RT-qPCR (Dog 36 in Table S1). This animal was naturally infected by the Delta (B.1.617.2) variant. Dog 36 tested positive by pVNT (ID50 884), ELISA-1 (%IH = 67.6%), and ELISA-2 (%IH = 73.1%), but negative by ELISA-3, with titers of N protein antibodies (S/P% = 49.3) close to the cut-off deeming it doubtful (S/P% = 50–60%). The serum sample was collected two months after the RT-qPCR positive result (Table S1). Regarding domestic goats, the experimental infection with the Beta variant (B.1.351) was confirmed by RT-qPCR in all cases [26]; three goats (Goat 1, Goat 2, and Goat 3 in Table S1) were sampled at 2 dpi, while the other three animals (Goat 4, Goat 5, and Goat 6 in Table S1) were sampled at 18 dpi. All goats from 2 dpi tested negative for RBD nAbs by ELISA-1, and for N protein antibodies by ELISA-3. Goats’ sera were not tested by ELISA-2. One goat from 2 dpi (Goat 3 in Table S1) tested positive by pVNT with titers of ID50 92.73, while the remaining ones tested negative. All goats from 18 dpi tested positive by both ELISA-1 and pVNT. With regard to the naturally infected lions, all tested positive by RT-qPCR (Pango lineage B.1.177) within 10 days (Lion 4 in Table S1), 24 days (Lion 1 and Lion 3 in Table S1), and 40 days (Lion 2 in Table S1) before the blood sampling [24]. Also, all lions tested positive by pVNT and for RBD nAbs by ELISA-1 and ELISA-2, but negative for N protein antibodies by ELISA-3. None of the wild boar were tested by RT-qPCR.

## 4. Discussion

Numerous serological assays have been developed to detect exposure to SARS-CoV-2 in humans [3,6,31]. Nevertheless, within the veterinary field, several serological tests have been adapted from those designed for humans without previous validation for animal samples [3]. The detection of acute SARS-CoV-2 infection in animal species faces more difficulties than in the human population. Most animals do not exhibit clinical signs upon SARS-CoV-2 infection, are often widely distributed (especially free-range wild animals, which complicates contact tracing), and sample accessibility can be complicated [1]. Thus, the validation of serological assays for animals is crucial for detecting SARS-CoV-2 antibodies, since it would help demonstrating viral exposure and potential susceptibility to infection [31,32].

Considering the interest in investigating SARS-CoV-2 humoral immune responses in animal species, the present study aimed to evaluate the correlation among three ELISAs kits (ELISA-1, ELISA-2 and ELISA-3) and compare them using the pVNT as the reference. ELISA-1 demonstrated the highest diagnostic performance, with a sensitivity of 96.9% for serum samples with pVNT titers > 60, compared to ELISA-2 (51.7–65.5%), and ELISA-3 (35.7%). Consequently, ELISA-1 offered the highest probability that animals testing negative truly lack nAbs. Importantly, ELISA-2 failed to detect positive samples with low nAbs titers (<1000), whereas ELISA-1 detected positive samples with pVNT titers as low as 60. Since the main objective of this study was to identify the most accurate kit for initial screenings, achieving high sensitivity was crucial. Thus, despite presenting a slightly lower specificity (97.1%) compared to the other tests (100.0%), ELISA-1 was the most suitable choice. This may be explained because ELISA-1 uses a lower cut-off value (%IH = 30%) than ELISA-2 (%IH = 35%) and ELISA-3 (S/P% = 60%). False-positive results could be confirmed with pVNT or other gold-standard tests like VNT or PRNT. Contrarily, ELISA-2 or ELISA-3 could potentially dismiss a significant number of seropositive samples.

ELISA-1 and ELISA-2 are both surrogate neutralization tests targeting RBD nAbs, although not all anti-RBD antibodies are necessarily neutralizing. Accordingly, pVNT also evaluates the neutralizing response against the S glycoprotein, where RBD is located [33]. Considering that the ELISA-3 detects N protein antibodies, lowest diagnostic performance, and lowest correlation with pVNT was already expected. Both ELISA-1 and ELISA-2 use purified, recombinant RBD protein and the host-cell receptor ACE2, evaluating the inhibition capacity of antibodies to neutralize RBD-ACE2 interaction [34]. This strategy gives clear advantages over conventional ELISAs that do not differentiate between nAbs and non-neutralizing binding Abs, together with the fact that ELISA-1 and ELISA-2 are isotype- and species independent [18,31]. Although these ELISAs are both competitive based, the design of each assay is different. ELISA-1 uses plates coated with the hACE2 extracellular domain and soluble RBD-HRP [3], whereas ELISA-2 uses pre-coated RBD plates and biotinylated ACE2 to dilute sera samples and further nAbs detection. The different orientation of the RBD and ACE2 proteins between kits may explain the differences observed between them.

A previous comparative study of various ELISAs testing human samples corroborated the higher sensitivity of ELISA-1 (93.7%) over ELISA-2 (56.4%) using the VNT as a reference [31]. All ELISAs included herein have already been used in previous studies to investigate humoral responses in both humans and animals [3,21,35,36,37,38]. However, both ELISA-1 and ELISA-2 were validated using human samples, and only ELISA-3 was validated for animal species including dogs, cats, cattle, horse, goat, and sheep. ELISA-3 used pre-pandemic samples for all mentioned animal species and exhibited an overall specificity of 99.1%, as described by the manufacturer’s protocol. Nevertheless, a few positive samples were included to test the sensitivity for each species. Moreover, serum samples from wildlife animals were not included in this validation, which could explain the differences in assay sensitivity and specificity across species. In the present study, we observed that sensitivity for dog samples was strongly reduced in ELISA-2 (with values of 16.7–33.3%) and ELISA-3 (with a value of 33.3%). For cat samples, ELISA-2 exhibited a sensitivity of 72.7–90.9% and ELISA-3 exhibited a sensitivity of 66.7%. ELISA-1 is still the most accurate kit for both species, with a sensitivity of 100%. Importantly, wildlife species are usually exposed to a major number of pathogens and their samples usually have lower quality than those from domestic animals [39]. Accordingly, samples from this group of species likely exhibit less specificity in serological tests.

Here, we detected two positive wild boar using ELISA-1 (with low percentage of inhibition), although they were negative by pVNT, ELISA-2, and ELISA-3. Both wild boar also tested negative by VNT [40]. In a previously published study, sera from five out of fourteen wild boar collected during the COVID-19 pandemic were able to weakly neutralize SARS-CoV-2 by VNT (titers of nAbs from 10 to 50) [41]. Although potential exposure to SARS-CoV-2 of this animal species may not be entirely ruled out, the low titers of nAbs supports that their sera components may yield false positive results in VNT [41]. We used a cut-off of ID50 = 60 for pVNT, which could have prevented potential false-positive results for this species and led to characterize the two ELISA-positive wild boar as negative for SARS-CoV-2 neutralization. Importantly, Hulst et al. [41] observed that the sera of juvenile pigs from the pre-pandemic period cross-reacted with recombinant S and N protein of SARS-CoV-2 by ELISA. These authors suspected that animals were previously infected with Alpha and Beta swine CoVs, which increased the likelihood of a potential cross-reactivity of antibodies. A previous study assessing SARS-CoV-2 infection in wildlife species also suggested cross-reactivity in sera from wild boar using ELISA-3, as they observed negative results when using ELISA-1 as a confirmatory test [42]. In the same line, another study confirmed two-way cross-reactivity for SARS-CoV and porcine CoV (transmissible gastroenteritis CoV-TGEV- and porcine respiratory CoV-PRCV) in ELISA, although it was demonstrated to be mediated by the N protein but not by the S glycoprotein [43]. Klompus et al. [44], investigating the cross-reactivity of SARS-CoV-2 with different peptides from animal CoVs also confirmed positive results regarding porcine CoV peptides. Altogether, it highlights the importance of establishing specific cut-offs for serological analysis for each animal species.

Despite the high specificity observed for ELISA-3 in our study, it is known that the N protein gene is highly conserved among CoVs and can potentially lead to the cross-reactivity of antibodies. Diezma-Díaz et al. [18] used pre-pandemic samples from cats and dogs and observed cross-reactivity by N-protein-based ELISAs. Other studies also suggested the cross-reactivity of N-protein antibodies from canine CoVs in serum samples from dogs, but also in serum samples from domestic cats previously infected by feline CoV (FCoV), using N-antigen ELISA assays [20,21,45]. Cross-reactivity to SARS-CoV-2 of antibodies targeting the RBD has been suggested in previous studies for some animal species, including domestic cats [18,44,46,47]. Importantly, the RBDs of SARS-CoV-2 exhibit higher antigenic distinctiveness from other animal CoVs compared to the N protein antigen or even when considering the whole S glycoprotein [18,20,28,48]. Previous studies using human samples already demonstrated cross-reactivity with SARS-CoV-2 of antibodies targeting the N protein of hCoVs, principally Alpha-hCoV (229E and NL363), but also Beta-hCoV (HKU1 and OC43) [13]. Technical cross-reactivity can be reduced using truncated versions from less conserved fragments of the N protein sequence instead of using the full length [13]. ELISA-3 is based on the truncated N recombinant antigen, which could affect the specificity and sensitivity of this test [13,45].

According to our results, previous studies supported that ELISAs based on the S glycoprotein and RBD protein are more accurate than those based on the N protein to diagnose seroconversion against SARS-CoV-2, especially in cats and dogs [18,20]. Diezma-Díaz et al. [18] found that ELISA-3 (targeting N protein antibodies) did not correlate with VNT and had a poor diagnostic performance (AUC = 0.55) for dog samples, and a weakly but significant correlation and better diagnostic performance (AUC = 0.90) when analyzing cat samples. Consistent with this, our data demonstrated that ELISA-3 had a lower sensitivity for dog samples (33.3%) than for cat samples (66.7%). Other studies did not find a correlation of ELISA-3 or other N-protein based assays with VNT or pVNT, neither for cats nor for dogs [20,21]. Regarding the human population, levels of N protein antibodies are positively correlated with COVID-19 severity [13]. Considering that dogs demonstrated lower susceptibility upon experimental and natural conditions compared to cats, they are likely developing lower antibody titers that could be dismissed by the low sensitivity of ELISA-3 [8,49].

Assay sensitivity may also change depending on the time from viral exposure or infection to sample collection, as it is known that measurable SARS-CoV-2 antibodies wane over time [50,51]. All pet animals included in this study were sampled owing to veterinary check-ups, and in some cases, due to clinical signs associated with SARS-CoV-2 infection. Despite evidence of viral exposure in some animals by the presence of nAbs, the timing between exposure and sampling was unknown. All pets tested negative for acute infection, except for one dog. This animal was sampled for antibody detection two months after acute infection, and exhibited positive neutralization by pVNT, with nAbs against RBD according to ELISA-1 and ELISA-2. The dog exhibited N protein antibodies within the limit to consider it as doubtful. These results could be explained by the fact that N protein antibodies are known to persist for a shorter duration compared to RBD or S glycoprotein nAbs, at least in the human population [51]. This was also the case of the naturally infected lions, which were sampled for antibody detection at least 10 days, and maximum 40 days after detection of acute infection, and did not exhibit N protein antibodies but RBD nAbs [24]. This fact could partially explain the negative results in ELISA-3 in most of the samples of this study, when the presence of nAbs was confirmed by the other assays. Animals could have been potentially exposed to the virus long before the time of sampling.

Our analyses are mainly limited by the lack of previous validation of pVNT for animal samples. For this purpose, positive and negative controls for each species of interest should be included to establish appropriate cut-off values for this test. Considering that cross-reactivity with other CoVs is not uncommon, the use of serum samples from animals previously infected with other CoVs, as well as samples from the pre-pandemic period may be beneficial to validate the pVNT. In addition, a larger number of samples from each species from the present comparative study may be included to assess sensitivity, specificity, and an appropriate cut-off for specific species. Considering the advantages of pVNT over the VNT or PRNT (e.g., time consuming, sample-processing capacity, more safety, and its use in BSL-2 facilities), the validation of pVNT for animal samples could be very useful to use it as a confirmatory test after previous ELISA screenings. The pVNT used here was developed and validated using human samples and the VNT as a reference. Interestingly, pVNT and VNT exhibited high qualitative and quantitative correlation (r [Spearman test] = 0.865) [6]. An additional trait of pVNT is its higher adaptability for testing newly emerging variants using pseudoviruses expressing the S glycoprotein of each variant of interest. In our study, all samples were tested with ELISAs that used the RBD of the ancestral (B.1 lineage) SARS-CoV-2, as well as the pVNT with the pseudotype expressing the S protein only from the ancestral virus. Thus, this analysis may be adapted using the RBD or the S glycoprotein of the newly Omicron variants and check whether there is a similar correlation. Those animals that tested negative might test positive for antibodies against the Omicron variant or its sub-lineages. However, only 12 out of 101 serum samples from this study were collected between December 2021 and February 2022, when the Omicron variant emerged in Spain. Despite indicated limitations, the comparative approach among three ELISA kits and pVNT in the context of multiple species still provides valuable insights, particularly in regards test performance variability.

## 5. Conclusions

ELISA-1 proved the most suitable kit for the initial screening of animal samples, particularly for cats and dogs. ELISA-3 may complement these screenings by detecting N-protein antibodies. ELISA-1 was shown to be not only effective but also efficient, requiring only ≈1.5 h from start to finish, while ELISA-2 and ELISA-3 required ≈2.5 h. However, despite ELISA-1’s high diagnostic capacity and efficiency, it cannot replace pVNT, which remains essential for quantifying nAb levels and assessing neutralization beyond the RBD.

## Figures and Tables

**Table 1:** Comparison of sensitivity and specificity of ELISA-1, ELISA-2, and ELISA-3 using the pVNT as a reference (cut-off = ID50 60). Seropositivity was defined by a cut-off of %IH ≥ 30% for ELISA-1, %IH ≥ 35% for ELISA-2, S/P% ≥ 60 for ELISA-3. P: positive; N: negative; PPV: positive predictive value; NPV: negative predictive value. The confidence interval (95%) is also represented for each parameter.

		pVNT						
		P	N		Sensitivity (%95 CI)	Specificity (%95 CI)	PPV (%95 CI)	NPV (%95 CI)
**ELISA 1**	**P**	32	2	34	96.90% (91.1–102.8%)	97.05% (93.0–101.1%)	94.10% (86.2–102.0%)	98.50% (50.6–101.4%)
	**N**	1	66	67				
		33	68	101				
**ELISA 2**	**P**	15	0	15	51.72% ^a^ (33.5–69.9%) 65.50% (48.2–82.8%)	100.00% (100.0–100.0%)	100.0% (100.0–100.0%)	80.60% ^b^ (71.4–89.7%) 85.30% (76.9–93.7%)
	**N**	14	58	72				
		29	58	87				
**ELISA 3**	**P**	12	0	12	38.70% (21.6–55.9%)	100.00% (100.0–100.0%)	100.00% (100.0–100.0%)	78.20% (69.5–6.8%)
	**N**	19	68	87				
		31	68	99				

^a^ The sensitivity and ^b^ NPV increased from 51.72% to 65.51% and from 80.60% to 85.30%, respectively, when doubtful samples (n=4) from ELISA-2 were considered positive.

**Table 2:** Comparison of sensitivity and specificity of ELISA-1, ELISA-2, and ELISA-3 using the pVNT as a reference (cut-off = ID50 60) in cats and dogs, independently. Seropositivity was defined by a cut-off of %IH ≥ 30% for ELISA-1, %IH ≥ 35% for ELISA-2, S/P% ≥ 60 for ELISA-3. P: positive; N: negative PPV: positive predictive value; NPV: negative predictive value. The confidence interval (95%) is also represented for each parameter.

			pVNT						
			P	N		Sensitivity (%95 CI)	Specificity (%95 CI)	PPV (%95 CI)	NPV (%95 CI)
CATS	**ELISA 1**	**P**	11	0	11	100% (100.0–100.0%)	100% (100.0–100.0%)	100% (100.0–100.0%)	100% (100.0–100.0%)
		**N**	0	25	25				
			11	2	36				
	**ELISA 2**	**P**	8	0	8	72.70% ^a^ (46.4–99.9%) 90.90% (73.9–107.9%)	100% (100.0–100.0%)	100% (100.0–100.0%)	89.3% ^b^ (77.8–100.7%) 96.2% (88.8–103.5%)
		**N**	3	25	28				
			11	25	36				
	**ELISA 3**	**P**	6	0	6	66.70% (35.9–97.5%)	100% (100.0–100.0%)	100% (100.0–100.0%)	89.3% (77.8–100.7%)
		**N**	3	25	28				
			9	25	34				
DOGS	**ELISA 1**	**P**	12	0	12	100% (100.0–100.0%)	100% (100.0–100.0%)	100% (100.0–100.0%)	100% (100.0–100.0%)
		**N**	0	29	29				
			12	29	41				
	**ELISA 2**	**P**	2	0	2	16.70% ^c^ (−4.4–37.8%) 33.30% (6.70–60.0%)	100% (100.0–100.0%)	100% (100.0–100.0%)	74.40% ^d^ (60.7–88.1%) 78.4% (65.1–91.6%)
		**N**	10	29	39				
			12	29	41				
	**ELISA 3**	**P**	4	0	4	33.30% (6.70–60.0%)	100% (100.0–100.0%)	100% (100.0–100.0%)	78.40% (65.10–91.6%)
		**N**	8	29	37				
			12	41	41				

^a,c^ Sensitivity and ^b,d^ NPV increased from 72.70% to 90.90% and 89.30% to 96.20%, respectively, for cats, and from 16.70% to 33.30% and 74.40% to 78.40%, respectively, for dogs, when doubtful samples (n = 2 for each species) in ELISA-2 were considered positive.

## Data Availability

All the data of this study are included in the manuscript and Table S1.

## Supplementary Materials

The following supporting information can be downloaded at: https://www.mdpi.com/article/10.3390/v17050716/s1, Table S1: Comparison of sensitivity and specificity of ELISA-1, ELISA-2 and ELISA-3 using the pVNT as a reference (Cut-off = ID50 60). Seropositivity was defined by a cut-off of %IH ≥30% for ELISA-1, % IH ≥35% for ELISA-2, S/P% ≥ 60 for ELISA-3.
