# Supplementary material for: Comparison of Three Commercial ELISA Kits for Detection of Antibodies Against SARS-CoV-2 in Serum Samples from Different Animal Species
Source: Viruses. 2025 May 16;17(5):716. doi: 10.3390/v17050716 (PMC12116064; doi:10.3390/v17050716)
Supplement: Supplementary file 1 [file viruses-17-00716-s001.zip › viruses-3607153-supplementary.pdf]

Table S1. Comparison of sensitivity and specificity of ELISA-1, ELISA-2 and ELISA-3 using the pVNT as a reference (Cut-off = ID_{50} 60). Seropositivity was defined by a cut-off of %IH ≥30% for ELISA-1, % IH ≥35% for ELISA-2, S/P% ≥ 60 for ELISA-3.

Species	Date of sampling	COVID-19 pandemic wave in Spain	ELISA-1 (%IH)	ELISA-2 (%IH)	ELISA-3 (S/P%)	pVNT ID_{50}	RT-qPCR
**Cat 1**	April 2020	Wuhan ancestral (B.1 lineage)	Positive 96.87	Positive 99.06	Positive 1393.43	Positive 6413	Negative
**Cat 2**	April 2020	Wuhan ancestral (B.1 lineage)	Positive 96.59	Positive 99.53	Positive 116.00	Positive 9315	Negative
**Cat 3**	May 2020	Wuhan ancestral (B.1 lineage)	Positive 96.87	Positive 99.19	NA	Positive 9570	Negative
**Cat 4**	June 2020	Wuhan ancestral (B.1 lineage)	Positive 96.67	Positive 99.53	Positive 1070.91	Positive 6051	Negative
**Cat 5**	May 2020	Wuhan ancestral (B.1 lineage)	Negative -14.68	Negative -6.89	Negative 0.38	Negative <60	Negative
**Cat 6**	May 2020	Wuhan ancestral (B.1 lineage)	Negative -19.27	Negative -27.91	Negative 2.21	Negative <60	Negative
**Cat 7**	June 2020	Wuhan ancestral (B.1 lineage)	Negative -16.65	Negative -15.69	Negative -4.05	Negative <60	Negative
**Cat 8**	January 2021	Alpha (B.1.1.7)	Positive 97.00	Positive 99.46	NA	Positive 5818	Negative
**Cat 9**	April 2021	Alpha (B.1.1.7)	Positive 96.06	Positive 98.46	Negative 6.36	Positive 2724	Negative
**Cat 10**	May 2021	Alpha (B.1.1.7)	Positive 96.83	Positive 99.19	Positive 263.45	Positive 3552	Negative
**Cat 11**	March 2021	Alpha (B.1.1.7)	Negative -26.81	Negative -22.81	Negative 0.10	Negative <60	Negative
**Cat 12**	April 2021	Alpha (B.1.1.7)	Negative -39.80	Negative -15.69	Negative 0.14	Negative <60	Negative
**Cat 13**	May 2021	Alpha (B.1.1.7)	Negative -55.62	Negative -10.31	Negative 2.07	Negative <60	Negative
**Cat 14**	June 2021	Alpha (B.1.1.7)/ Delta (B.1.617.2)	Positive 35.15	Negative 19.72	Negative 19.64	Positive 438	Negative
**Cat 15**	August 2021	Delta (B.1.617.2)	Positive 34.2	Doubtful 29.33	Negative 12.73	Positive 189	Negative
**Cat 16**	August 2021	Delta (B.1.617.2)	Negative 12.81	Negative -5.48	Negative 1.10	Negative <60	Negative
**Cat 17**	November 2021	Delta (B.1.617.2)	Negative 16.54	Negative -13.34	Negative 0.05	Negative <60	Negative
**Cat 18**	September 2021	Delta (B.1.617.2)	Negative -2.415	Negative -17.90	Negative 0.00	Negative <60	Negative
**Cat 19**	September 2021	Delta (B.1.617.2)	Negative 15.79	Negative -19.38	Negative 1.44	Negative <60	Negative
**Cat 20**	September 2021	Delta (B.1.617.2)	Negative 9.57	Negative -21.8	Negative -0.05	Negative <60	Negative

**Cat 21**	November<br>2021	Delta<br>(B.1.617.2)	Negative<br>14.24	Negative<br>-24.02	Negative<br>-0.10	Negative<br><60	Negative
**Cat 22**	November<br>2021	Delta<br>(B.1.617.2)	Negative<br>-10.85	Negative<br>-15.96	Negative<br>0.05	Negative<br><60	Negative
**Cat 23**	November<br>2021	Delta<br>(B.1.617.2)	Negative<br>-20	Negative<br>-22.34	Negative<br>1.54	Negative<br><60	Negative
**Cat 24**	November<br>2021	Delta<br>(B.1.617.2)	Negative<br>10.65	Negative<br>-23.68	Negative<br>0.48	Negative<br><60	Negative
**Cat 25**	November<br>2021	Delta<br>(B.1.617.2)	Negative<br>9.73	Negative<br>-24.03	Negative<br>0.05	Negative<br><60	Negative
**Cat 26**	November<br>2021	Delta<br>(B.1.617.2)	Negative<br>2.13	Negative<br>-9.57	Negative<br>2.93	Negative<br><60	Negative
**Cat 27**	November<br>2021	Delta<br>(B.1.617.2)	Negative<br>-1.59	Negative<br>-15.08	Negative<br>-0.31	Negative<br><60	Negative
**Cat 28**	November<br>2021	Delta<br>(B.1.617.2)	Negative<br>5.68	Negative<br>-15.08	Negative<br>0.05	Negative<br><60	Negative
**Cat 29**	November<br>2021	Delta<br>(B.1.617.2)	Negative<br>11.78	Negative<br>-16.90	Negative<br>0.63	Negative<br><60	Negative
**Cat 30**	December<br>2021	Delta<br>(B.1.617.2)/<br>Omicron (BA.1)	Positive<br>92.15	Doubtful<br>29.93	Positive<br>110.36	Positive<br>682	Negative
**Cat 31**	December<br>2021	Delta<br>(B.1.617.2)/<br>Omicron (BA.1)	Negative<br>8.23	Negative<br>-19.25	Negative<br>0.05	Negative<br><60	Negative
**Cat 32**	December<br>2021	Delta<br>(B.1.617.2)/<br>Omicron (BA.1)	Negative<br>3.63	Negative<br>21.93	Negative<br>-0.19	Negative<br><60	Negative
**Cat 33**	December<br>2021	Delta<br>(B.1.617.2)/<br>Omicron (BA.1)	Negative<br>12.36	Negative<br>2.05	Negative<br>0.62	Negative<br><60	Negative
**Cat 34**	December<br>2021	Delta<br>(B.1.617.2)/<br>Omicron (BA.1)	Negative<br>2.88	Negative<br>-4.00	Negative<br>0.34	Negative<br><60	Negative
**Cat 35**	December<br>2021	Delta<br>(B.1.617.2)/<br>Omicron (BA.1)	Negative<br>4.43	Negative<br>-12.19	Negative<br>0.24	Negative<br><60	Negative
**Cat 36**	January<br>2022	Omicron (BA.1)	Positive<br>89.93	Positive<br>93.95	Positive<br>544.73	Positive<br>2388	Negative
**Dog 1**	July<br>2020	Wuhan ancestral<br>(B.1 lineage)	Positive<br>51.07	Doubtful<br>28.92	Negative<br>9.82	Positive<br>601	Negative
**Dog 2**	December<br>2020	Wuhan ancestral<br>(B.1 lineage)	Positive<br>52.87	Doubtful<br>30.60	Negative<br>-55.00	Positive<br>702	Negative
**Dog 3**	May<br>2020	Wuhan ancestral<br>(B.1 lineage)	Negative<br>29.53	Negative<br>-1.92	Negative<br>0.10	Negative<br><60	Negative
**Dog 4**	August<br>2020	Wuhan ancestral<br>(B.1 lineage)	Negative<br>-3.38	Negative<br>0.64	Negative<br>0.53	Negative<br><60	Negative
**Dog 5**	March<br>2020	Alpha (B.1.1.7)	Negative<br>7.09	Negative<br>-7.63	Negative<br>0.29	Negative<br><60	Negative
**Dog 6**	March<br>2020	Alpha (B.1.1.7)	Negative<br>-2.69	Negative<br>-20.86	Negative<br>0.24	Negative<br><60	Negative
**Dog 7**	April<br>2021	Alpha (B.1.1.7)	Negative<br>-12.22	Negative<br>-15.62	Negative<br>0.34	Negative<br><60	Negative

**Dog 8**	April<br>2021	Alpha (B.1.1.7)	Negative<br>5.11	Negative<br>-6.75	Negative<br>2.31	Negative<br><60	Negative
**Dog 9**	April<br>2021	Alpha (B.1.1.7)	Negative<br>7.11	Negative<br>-10.65	Negative<br>3.17	Negative<br><60	Negative
**Dog 10**	April<br>2021	Alpha (B.1.1.7)	Negative<br>-6.04	Negative<br>-6.15	Negative<br>0.96	Negative<br><60	Negative
**Dog 11**	April<br>2021	Alpha (B.1.1.7)	Negative<br>4.80	Negative<br>-9.91	Negative<br>-0.05	Negative<br><60	Negative
**Dog 12**	April<br>2021	Alpha (B.1.1.7)	Negative<br>-8.64	Negative<br>-15.55	Negative<br>0.34	Negative<br><60	Negative
**Dog 13**	April<br>2021	Alpha (B.1.1.7)	Negative<br>7.95	Negative<br>-19.65	Negative<br>0.34	Negative<br><60	Negative
**Dog 14**	April<br>2021	Alpha (B.1.1.7)	Negative<br>13.37	Negative<br>-20.93	Negative<br>3.55	Negative<br><60	Negative
**Dog 15**	April<br>2021	Alpha (B.1.1.7)	Negative<br>2.61	Negative<br>-13.81	Negative<br>0.14	Negative<br><60	Negative
**Dog 16**	May<br>2021	Alpha (B.1.1.7)	Negative<br>14.10	Negative<br>-9.84	Negative<br>1.68	Negative<br><60	Negative
**Dog 17**	May<br>2021	Alpha (B.1.1.7)	Negative<br>1.40	Negative<br>-13.74	Negative<br>0.19	Negative<br><60	Negative
**Dog 18**	May<br>2021	Alpha (B.1.1.7)	Negative<br>4.12	Negative<br>-15.08	Negative<br>0.14	Negative<br><60	Negative
**Dog 19**	April<br>2021	Alpha (B.1.1.7)	Negative<br>-2.25	Negative<br>-2.72	Negative<br>2.45	Negative<br><60	Negative
**Dog 20**	May<br>2021	Alpha (B.1.1.7)	Negative<br>13.81	Negative<br>-8.50	Negative<br>0.58	Negative<br><60	Negative
**Dog 21**	May<br>2021	Alpha (B.1.1.7)	Negative<br>9.17	Negative<br>-3.53	Negative<br>0.05	Negative<br><60	Negative
**Dog 22**	May<br>2021	Alpha (B.1.1.7)	Negative<br>0.96	Negative<br>-29.26	Negative<br>2.46	Negative<br><60	Negative
**Dog 23**	April<br>2021	Alpha (B.1.1.7)	Negative<br>5.19	Negative<br>-12.53	Negative<br>0.11	Negative<br><60	Negative
**Dog 24**	April<br>2021	Alpha (B.1.1.7)	Negative<br>9.98	Negative<br>-7.09	Negative<br>0.42	Negative<br><60	Negative
**Dog 25**	April<br>2021	Alpha (B.1.1.7)	Negative<br>-3.76	Negative<br>-7.02	Negative<br>0.26	Negative<br><60	Negative
**Dog 26**	April<br>2021	Alpha (B.1.1.7)	Negative<br>-10.24	Negative<br>-7.36	Negative<br>0.52	Negative<br><60	Negative
**Dog 27**	April<br>2021	Alpha (B.1.1.7)	Negative<br>2.61	Negative<br>-2.05	Negative<br>0.42	Negative<br><60	Negative
**Dog 28**	May<br>2021	Alpha (B.1.1.7)	Negative<br>-13.30	Negative<br>-18.37	Negative<br>0.19	Negative<br><60	Negative
**Dog 29**	April<br>2021	Alpha (B.1.1.7)	Negative<br>-13.15	Negative<br>-7.02	Negative<br>0.24	Negative<br><60	Negative
**Dog 30**	June<br>2021	Alpha (B.1.1.7)/<br>Delta<br>(B.1.617.2)	Positive<br>30.62	Negative<br>-5.34	Positive<br>861.82	Positive<br>519	Negative
**Dog 31**	June<br>2021	Alpha (B.1.1.7)/<br>Delta<br>(B.1.617.2)	Positive<br>85.51	Positive<br>82.40	Negative<br>21.82	Positive<br>5599	Negative
**Dog 32**	June	Alpha (B.1.1.7)/	Negative	Negative	Negative	Negative	Negative

	2021	Delta (B.1.617.2)	-3.32	-21.53	0.00	<60	
**Dog 33**	July 2021	Delta (B.1.617.2)	Positive 36.06	Negative 17.77	Negative 4.73	Positive 296	Negative
**Dog 34**	September 2021	Delta (B.1.617.2)	Positive 34.35	Negative -17.64	Negative 43.27	Positive 974	Negative
**Dog 35**	September 2021	Delta (B.1.617.2)	Positive 37.22	Negative 1.11	Negative 38.55	Positive 436	Negative
**Dog 36**	28/09/2021	Delta (B.1.617.2)	Positive 67.55	Positive 73.13	Negative 49.27	Positive 884	Positive 27/07/2021
**Dog 37**	October 2021	Delta (B.1.617.2)	Positive 59.31	Negative 16.83	Negative 48.91	Positive 926	Negative
**Dog 38**	November 2021	Delta (B.1.617.2)	Positive 40.62	Negative 14.28	Negative 116.10	Positive 386	Negative
**Dog 39**	November 2021	Delta (B.1.617.2)	Positive 36.54	Negative 5.14	Positive 1238.55	Positive 378	Negative
**Dog 40**	July 2021	Delta (B.1.617.2)	Positive 62.21	Negative 4.27	Negative 617.82	Positive 166	Negative
**Dog 41**	August 2021	Delta (B.1.617.2)	Negative -5.48	Negative -15.62	Negative 0.77	Negative <60	Negative
**Ferret 1**	December 2020	Wuhan ancestral (B.1 lineage)	Negative 5.74	Negative -28.45	Negative 1.59	Negative <60	Negative
**Ferret 2**	December 2020	Wuhan ancestral (B.1 lineage)	Negative -1.61	Negative -27.24	Negative 1.39	Negative <60	Negative
**Ferret 3**	July 2021	Delta (B.1.617.2)	Positive 89.50	Positive 94.69	Positive 702.91	Positive 1247	Negative
**Ferret 4**	July 2021	Delta (B.1.617.2)	Positive 74.38	Negative 18.374	Positive 1145.45	Positive 767	Negative
**Goat 1**	2dpi	experimental / Beta (B.1.351)	Negative 14.71	NA	Negative -0.26	Negative <60	Positive
**Goat 2**	2dpi	experimental / Beta (B.1.351)	Negative 24.15	NA	Negative 3.97	Negative <60	Positive
**Goat 3**	2dpi	experimental / Beta (B.1.351)	Negative 20.72	NA	Negative 1.25	Positive 92.73	Positive
**Goat 4**	18 dpi	experimental / Beta (B.1.351)	Positive 59.59	NA	Negative 1.41	Positive 228.12	Positive
**Goat 5**	18 dpi	experimental / Beta (B.1.351)	Positive 41.45	NA	Negative 5.28	Positive 525.52	Positive
**Goat 6**	18 dpi	experimental / Beta (B.1.351)	Positive 34.70	NA	Negative 15.48	Positive 99.78	Positive
**Lion 1**	02.12.2020	Natural infection B.1.177	Positive 89.00	Positive 99.13	Negative 2.09	Positive 8769.50	Positive 09.11.2020
**Lion 2**	18.12.2020	Natural infection B.1.177	Positive 90.00	Positive 96.78	Negative 18.36	Positive 5491.64	Positive 09.11.2020
**Lion 3**	02.12.2020	Natural infection B.1.177	Positive 86.00	Positive 79.51	Negative 15.27	Positive 1224.93	Positive 09.11.2020
**Lion 4**	19.11.2020	Natural infection B.1.177	Positive 89.00	Positive 94.49	Negative 25.36	Positive 6238.82	Positive 09.11.2020
**Wild boar 1**	April 2020	Wuhan ancestral (B.1 lineage)	Negative 5.30	Negative NA	Negative -0.10	Negative <60	NA

**Wild boar 2**	November 2020	Wuhan ancestral /Alpha (B.1.1.7)	Negative 18.63	NA	Negative 2.56	Negative <60	NA
**Wild boar 3**	November 2020	Wuhan ancestral /Alpha (B.1.1.7)	Negative -10.11	NA	Negative 1.62	Negative <60	NA
**Wild boar 4**	May 2021	Alpha (B.1.1.7)	Positive 34.87	Negative -25.76	Negative 19.40	Negative <60	NA
**Wild boar 5**	August 2021	Delta (B.1.617.2)	Positive 34.87	Negative -20.32	Negative -0.36	Negative <60	NA
**Wild boar 6**	December 2021	Delta (B.1.617.2)/ Omicron (BA.1)	Negative 11.74	Negative NA	Negative -0.05	Negative <60	NA
**Wild boar 7**	December 2021	Delta (B.1.617.2)/ Omicron (BA.1)	Negative 8.88	Negative NA	Negative 0.10	Negative <60	NA
**Wild boar 8**	December 2021	Delta (B.1.617.2)/ Omicron (BA.1)	Negative 3.48	NA	Negative 0.68	Negative <60	NA
**Wild boar 9**	February 2022	Omicron (BA.1)	Negative 10.03	NA	Negative 0.10	Negative <60	NA
**Wild boar 10**	February 2022	Omicron (BA.1)	Negative 7.34	NA	Negative 0.37	Negative <60	NA
